# Internal Iliac Artery Embolization for the Control of Severe Bladder Hemorrhage Secondary to Carcinoma: Long-Term Follow-Up

**DOI:** 10.1100/tsw.2007.229

**Published:** 2007-09-17

**Authors:** Ahmed El-Assmy, Tarek Mohsen

**Affiliations:** ^1^ Urology Department, Urology and Nephrology Center, Mansoura University, Mansoura, Egypt; ^2^ Radiology Department, Urology and Nephrology Center, Mansoura University, Mansoura, Egypt

**Keywords:** embolization, internal iliac artery, neoplasms, bladder, hemorrhage

## Abstract

The purpose of this study was to evaluate the efficacy and long-term complications of internal iliac artery embolization as a palliative measure in the control of intractable hemorrhage from advanced bladder malignancy. From January 1998 through December 2005, seven patients underwent transcatheter arterial embolization (TAE) of anterior division of internal iliac artery bilaterally for intractable bladder hemorrhage. After embolization, patients were followed for the efficacy of the procedure in controlling hematuria and complications. TAE was successful in immediate control of severe hemorrhage in all seven patients after a mean period of 4 days. At a mean (range) follow-up of 10 (6–12) months, the hemorrhage was permanently controlled in four (57%) patients. Three patients developed hematuria and required emergency admissions; two had mild hematuria and were managed conservatively, and the remaining one required a second attempt of embolization after 2 months from the first one. During the whole period of follow-up, there were no significant complications related to embolization. Internal iliac artery embolization is an effective and minimally invasive option when managing advanced bladder malignancies presenting with intractable bleeding. The long-term follow-up showed control of bleeding in the majority of such patients with no serious complications.

## INTRODUCTION

Severe hemorrhage from an advanced bladder tumor can be life threatening. Methods of management for uncontrolled bladder bleeding include cystoscopic clot evacuation with cystodiathermy, Helmstein balloon compression[1], irrigation with alum solution[2], and instillation of formalin[3], and may finally culminate in open surgical techniques ranging from simple packing of the bladder to cystectomy and urinary diversion[4]. Most patients in this situation are elderly and unfit, and therefore unlikely to withstand these latter morbid procedures.

There has been a continuing search for a minimally invasive, less morbid procedure to be offered to these risky patients. Since the introduction of internal iliac embolization in 1974 by Hald and Myging[5], it has been successfully used in controlling bladder hemorrhage from terminal pelvic malignancy[6,7,8,9,10,11,12,13,14]. Some of these studies were case reports; others included nonhomogenous groups of patients. In addition, most of them lacked long-term follow-up.

This study was conducted for evaluation of the long-term efficacy of transcatheter arterial embolization (TAE) of bilateral internal iliac arteries in managing uncontrollable hemorrhage from advanced bladder neoplasms. We tried to overcome the limitations of the previous studies by providing a single institutional study containing a homogeneous group of patients (advanced bladder carcinoma) with long-term follow-up.

## MATERIALS AND METHODS

### Patients

From January 1998 through December 2005, seven patients underwent TAE of bilateral internal iliac arteries for intractable bladder hemorrhage. Six patients had advanced transitional cell carcinoma (TCC) of the urinary bladder and the remaining patient had advanced squamous cell carcinoma (SSC). All the patients had conservative treatment before TAE, which included continuous bladder irrigation using a three-way catheter and attempts to control bleeding endoscopically.

### Technique of Embolization

All embolization procedures were performed by one experienced interventional radiologist. The procedure started with intra-arterial digital subtraction angiography (IA-DSA) of the aorta followed by selective catheterization of the internal iliac artery. The anterior division of internal iliac artery was embolized using Platinum microcoils of 0.035 in. in all patients (Boston Scientific, Boston, MA) delivered through the 6 French (F) angiographic catheter (Copra II, Terumo, Osaka, Japan)(Figs. 1 and 2). In two patients, alcohol was used in addition to microcoils for embolization. Embolization with absolute alcohol was carried out using 7 French polyethylene Torcon balloon catheter after subselective catheterization of the branch to be occluded. The balloon was then inflated before the alcohol was injected to prevent reflux of the alcohol. The alcohol was slowly injected by hand at a rate of approximately 0.1–0.2 ml/sec. A test injection, at the same rate, was first performed to confirm the absence of reflux. Each injection was followed by infusion of 1 ml of saline at the same rate. After 10 min, the catheter was slowly withdrawn and aspirated, after deflation of the balloon, to remove possible residual alcohol and debris.

The technical success of the procedure was documented by doing angiography at the end of the embolization procedure (Fig. 3). The same procedure was repeated on the opposite side using an ipsilateral or contralateral puncture.

### Follow-Up

After embolization, patients were followed for symptoms of embolization syndrome, other complications of the procedures, and the efficacy of the procedure in controlling hematuria.

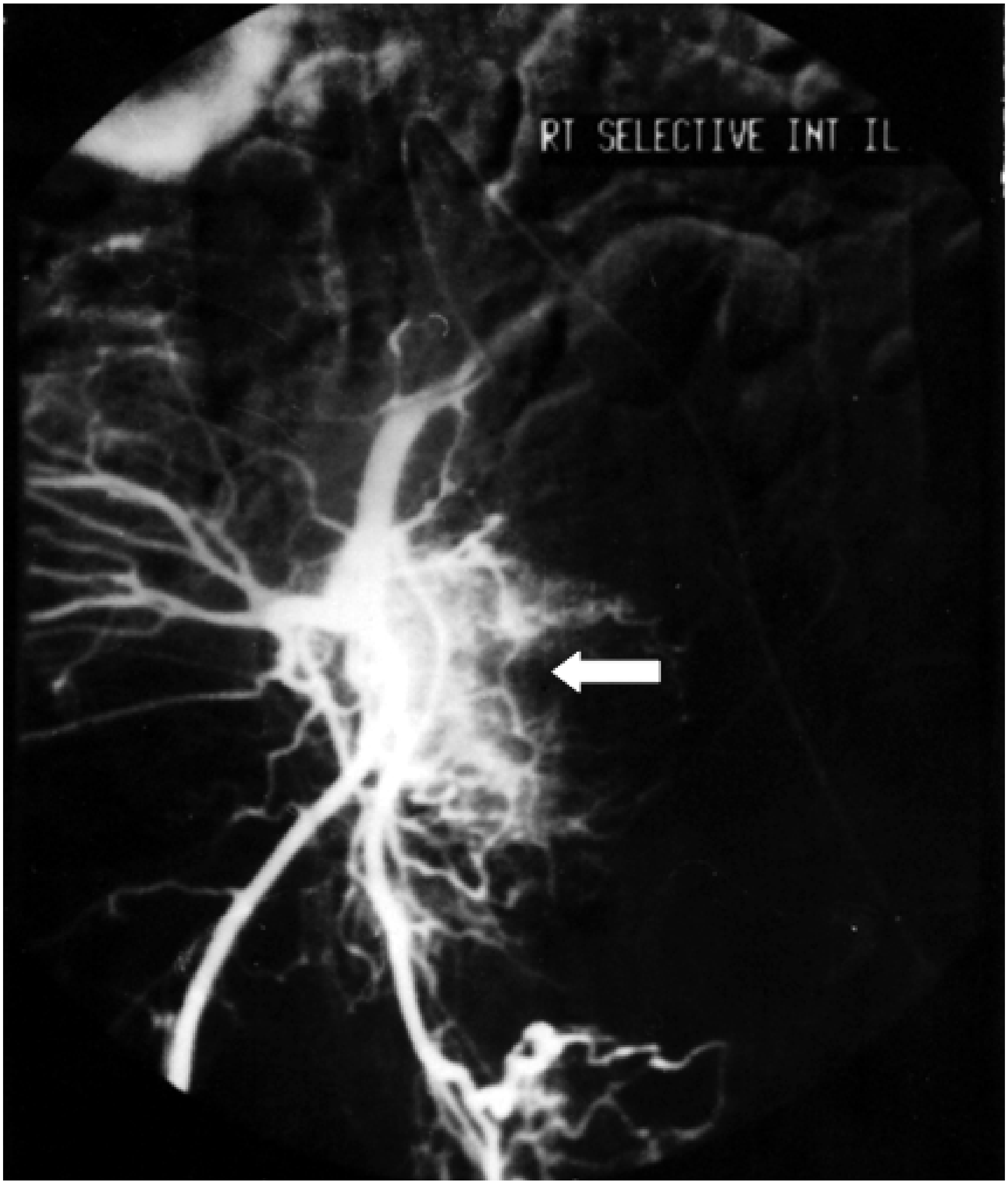

A

**FIGURE 1.** Pre-embolization subtraction arteriogram showing selective contralateral catheterization of the right (A) and left (B) internal iliac artery with significant vascular supply to the bladder wall tumor.

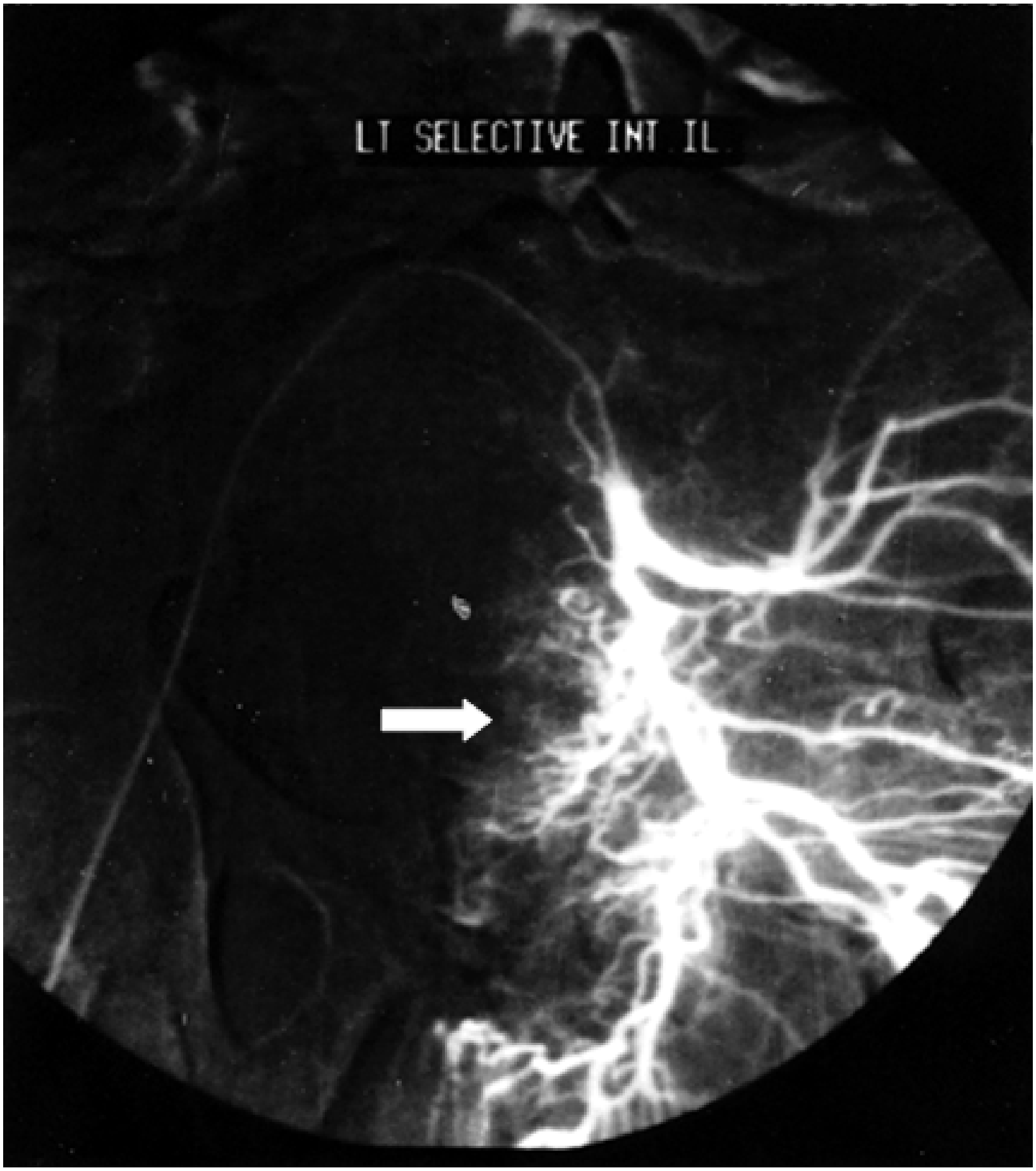

**FIGURE 1B**

## RESULTS

Our study included seven patients (sex male and one female) with a mean age of 61 (55–68 years), with severe hematuria due to advanced bladder neoplasms. Those patients were not candidates for surgical treatment because of metastatic disease in five patients and old age with bad general condition in the remaining two. Before embolization, the patients had required a mean (range) of 5 (2–8) emergency admissions and required 4.5 (4–7) blood transfusion units. Two patients with TCC had palliative radiotherapy to control the bleeding. The hematuria recurred in both at a mean (range) of 5 (6–8) months.

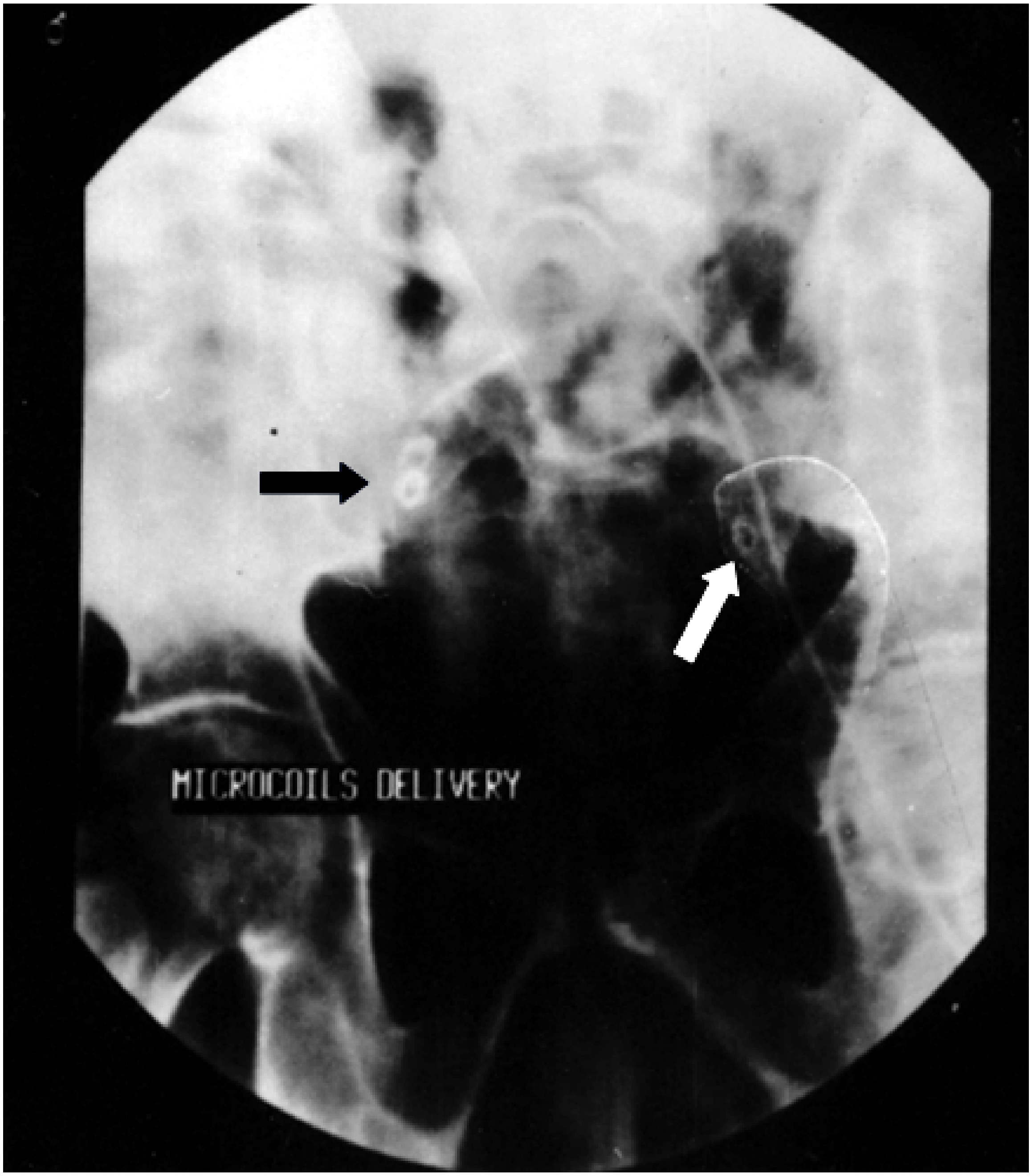

**FIGURE 2.** Postembolization subtraction arteriogram showing occlusion coils bilaterally within the anterior divisions of the internal iliac arteries.

TAE was successful in immediate control of severe hemorrhage in all seven patients after a mean period of 4 days. At a mean (range) follow-up of 10 (6–12) months, the hemorrhage was permanently controlled in four (57%) patients. Three patients developed hematuria and required emergency admissions (1.5, 2, and 2 months after embolization). Two of them were managed conservatively and one of them required a second attempt of embolization; those patients required 2.1 (2–4) transfusion units. During the whole period of follow-up, the later three patients did not develop other attacks of hematuria. Also, there were no significant complications related to embolization.

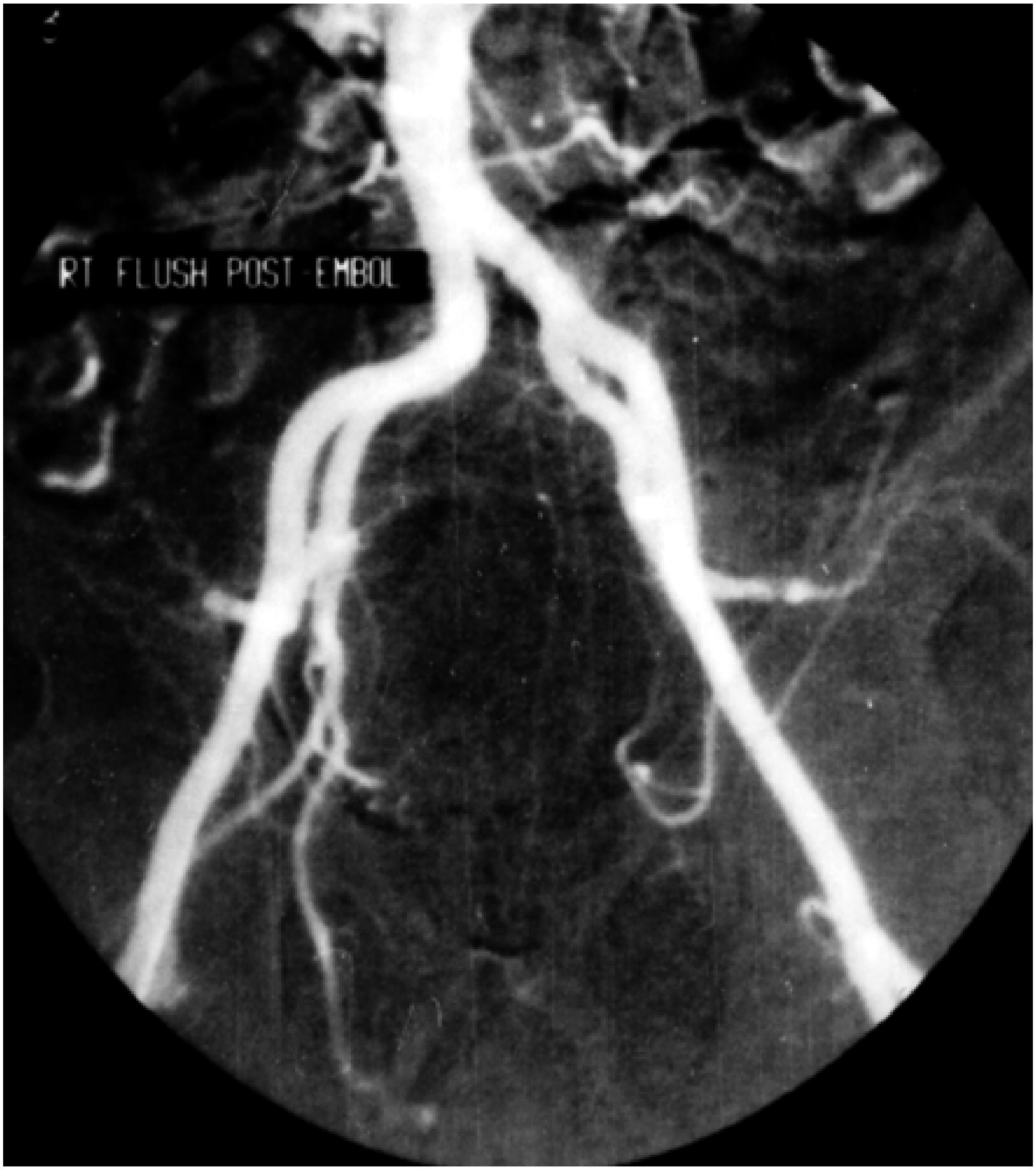

**FIGURE 3.** Postembolization subtraction arteriogram showing complete obliteration of blood flow to the bladder wall tumor.

## DISCUSSION

In clinical practice, most hematuria episodes secondary to advanced bladder malignancy are controlled by an effective continuous normal saline irrigation using three-way catheters and by endoscopic cauterization of bleeders. When these measures fail to control bleeding, the urologist faces a difficult clinical problem. Therapeutic embolization offers an alternative to surgical intervention in these patients.

Embolization has been used successfully to manage intractable bleeding in advanced pelvic malignancies, particularly gynecological tumors. Understandably, the threshold for embolization of gynecological tumors is high, as there are few options for intracavitary irrigation or balloon tamponade using a Foley catheter as a conservative measure. Pisco et al.[6], in a large review, reported complete control of bleeding in 69% of cases with pelvic malignancies by embolizing the anterior division of anterior iliac artery. However, their study included nonhomogenous groups of patients with gynecologic and urologic tumors. Also, there was no mention of follow-up of urological malignancies in that study.

There are reports of the successful control of hematuria after embolization in bladder and prostate cancers[6,7,8,9,10,11,12,13,14]. Appelton and associates[13] reported their experience with embolization of the internal iliac artery in eight patients with severe bladder hemorrhage and in two with severe bleeding from the prostatic bed after prostatectomy. Effective control of the bleeding was achieved in six of the patients with bladder hemorrhage. Both patients with postprostatectomy bleeding responded well to embolization. Nabi et al.[14] reported successful palliative management of intractable hematuria due to bladder and prostatic tumor by embolization of anterior division of internal iliac artery in six patients with no recurrence at a mean follow-up of 22 months. Gujral et al.[11] suggested an extended role of this procedure in the perioperative phase to control intractable bleeding following transurethral resection of bladder tumor. In our study, TAE of internal iliac arteries could stop bleeding immediately in four of seven (57%) patients. Even in the remaining three patients with recurrent hematuria, the attacks were less severe and could be managed conservatively in two of them, and only one required another attempt of embolization.

The anterior division of the internal iliac artery should be embolized bilaterally irrespective of whether the bleeding point is detectable on angiography or not[15]. This prevents the recurrence of bleeding from collaterals. The partial physiological alterations in mean pressure, pulse volume, and blood flow even after bilateral internal iliac embolization obviates the fear of ischemic infarction[16].

The most worrisome complications of TAE of internal iliac arteries are ischemic infarction and recurrence of bleeding. In the earlier study by Pisco et al.[6], TAE of the internal iliac arteries was performed in 108 patients with uncontrollable hemorrhage due to pelvic neoplasms (urinary bladder in 50, uterus in 39, ovary in 16, and prostate in three). The bleeding recurred in 33 patients, 70 patients experienced postembolization syndrome (nausea, vomiting, gluteal pain, and fever due to tissue necrosis), and three had transient acute tubular necrosis caused by the contrast medium. More recently in the study Nabi et al.[14], there were minor complications, e.g., nausea, vomiting, and fever in 50% of their patients, for a mean of 2 days and there was no major complication.

The mean follow-up in our study was 10 months, which is less than 22 months reported by Nabi et al.[14]. This may be due to the fact that half of the patients in their study were having prostatic adenocarcinoma and those patients tend to have a longer survival than our patients with advanced bladder carcinoma. The long-term follow-up in our study showed no complications, in contrast to previously reported series[6,15,16,17] and in agreement with that of Nabi et al.[14].

We used coils to permanently embolize the anterior division of internal iliac arteries. Careful radiographic visualization of the anterior division of internal iliac artery, precise placement of the coils, and the use of coils instead of particulate embolizing agents, to preserve tissue perfusion as these remain proximal to arterioles and do not interfere with capillary circulation[14], are probably important in minimizing complications.

## CONCLUSIONS

TAE of bilateral internal iliac arteries is an effective method for managing intractable hemorrhage in advanced bladder malignancy. The long-term follow-up showed good control of bleeding in the majority of such patients with no serious complications and decreased the requirement for blood transfusion rates. We recommend this technique for the control of severe bleeding from the bladder in advanced tumors or seriously ill patients.
